# Semiconductor-Ionic Nanocomposite La_0.1_Sr_0.9_MnO_3−δ_-Ce_0.8_Sm_0.2_O_2−δ_ Functional Layer for High Performance Low Temperature SOFC

**DOI:** 10.3390/ma11091549

**Published:** 2018-08-28

**Authors:** Zhaoqing Wang, Xunying Wang, Zhaoyun Xu, Hui Deng, Wenjing Dong, Baoyuan Wang, Chu Feng, Xueqi Liu, Hao Wang

**Affiliations:** Hubei Collaborative Innovation Center for Advanced Organic Chemical Materials, Hubei Key Laboratory of Ferro & Piezoelectric Materials and Devices, Faculty of Physics and Electronic Science, Hubei University; Wuhan 430062, Hubei, China; jackie925wang@163.com (Z.W.); 51161213054@stu.ecnu.edu.cn (Z.X.); denghui_135@sina.com (H.D.); wenjingd@hubu.edu.cn (W.D.); baoyuanw@163.com (B.W.); fc841373009@163.com (C.F.); lxq_9388@sina.com (X.L.)

**Keywords:** LT-SOFC, LSM (La_0.1_Sr_0.9_MnO_3−δ_), hetero-interface, semiconductor-ionic nanocomposite, junction

## Abstract

A novel composite was synthesized by mixing La_0.1_Sr_0.9_MnO_3−δ_ (LSM) with Ce_0.8_Sm_0.2_O_2−δ_ (SDC) for the functional layer of low temperature solid oxide fuel cell (LT-SOFC). Though LSM, a highly electronic conducting semiconductor, was used in the functional layer, the fuel cell device could reach OCVs higher than 1.0 V without short-circuit problem. A typical diode or rectification effect was observed when an external electric force was supplied on the device under fuel cell atmosphere, which indicated the existence of a junction that prevented the device from short-circuit problem. The optimum ratio of LSM:SDC = 1:2 was found for the LT-SOFC to reach the highest power density of 742 mW·cm^−2^ under 550 °C The electrochemical impedance spectroscopy data highlighted that introducing LSM into SDC electrolyte layer not only decreased charge-transfer resistances from 0.66 Ω·cm^2^ for SDC to 0.47–0.49 Ω·cm^2^ for LSM-SDC composite, but also decreased the activation energy of ionic conduction from 0.55 to 0.20 eV.

## 1. Introduction

Solid oxide fuel cells (SOFCs) research and development have been strongly driven towards low temperature (LT), <600 °C, operations. Thereby, the materials and technology associated with LT demand extensive research efforts. Among the three basic components of SOFC, the electrolyte is the core. Yttria-stabilized zirconia (YSZ) has dominated the SOFC R&D over many decades since it was first discovered by Nernst [1] in 1899. However, its sufficient O^2−^ conductivity, ~0.1 S cm^−1^, only takes place under high temperature (~1000 °C) [2,3], leading to high system costs and complexity, severe drawback on material durability, slow start-up rate, thus impeding the SOFCs commercial process. Therefore, the LTSOFC research and development have become worldwide, but to maintain the high efficiency and to design high ionic conductive electrolyte materials functioned at low temperatures, have been a critical challenge [4,5,6,7,8].

For developing new oxide-ion conducting materials aiming to replace YSZ as the electrolyte for LTSOFCs, the most common way is the structural design by doping to create structural defects, thus increasing ionic conductivity. Goodenough [9] proposed that the conductivity of oxide-ion conductors could be effectively enhanced by designing materials structure. For example, doping low valent cations can enhance the ionic conductivity by introducing oxygen vacancies. Although various electrolyte materials have been discovered following this line, there have been no alternative materials so far. Doped ceria possesses excellent O^2−^ conductivity (0.1 S cm^−1^) under 800 °C, 200 °C below than that of the YSZ [10,11,12]. However, the electronic conduction characteristic, owing to the Ce^4+^ to Ce^3+^ reduction in fuel cell operation, leading to losses for open circuit voltage (OCV) of the SOFC with doped ceria electrolytes [13].

Based on the previous studies, a novel way to develop effectively ionic conducting electrolytes by constructing more interfaces between the two-phase composite materials has been developed. At the hetero-interfacial region, highly disordered microstructure provides a large number of structural defects which can provide fast conducting pathways [2,14,15,16,17,18].

Zhu et al. [19,20,21,22,23,24,25,26,27] designed nano-composite two-phase materials with super ionic conduction by constructing hetero-interface with super ionic conduction, honored as ‘ion highway’ [28]. They significantly enhanced the material conductivity under lower temperature. Barriocanal et al. [2] fabricated hetero-structures by sandwiching YSZ electrolyte layers with different thicknesses between two layers of semiconducting SrTiO_3_ (STO). They found that the interface between hetero-structures (e.g., YSZ/STO) supplied both higher carrier concentration and lower activation energy of ionic conduction, thus yielding many orders to increase their ionic conductivity. Yao et al. [14] enhanced the ionic conductivity of single-layered SDC film about 5 times by constructing Ce_0.8_Sm_0.2_O_2−δ_/Al_2_O_3_ multilayer structure, demonstrating that increasing hetero-interface is beneficial to enhance ionic interfacial conductivity. According to their study results, the higher interfacial conductivity of Ce_0.8_Sm_0.2_O_2−δ_/Al_2_O_3_ multilayer structure is due to more structural defects at interfacial regions with highly-disordered microstructures. However, an important materials nature is missed. All of these heterostructures are constructed between the ionic conductor and the semiconductor, so it is more appropriate to name it as the semiconductor-ionic heterostructure. Based on this line, we can explore many new opportunities. Some of them have been reported [29,30,31], but there is a great potential for more research avenues to be explored.

Considering the high O^2−^ conductivity of SDC between 500 and 1000 °C and its good compatibility with Lanthanum strontium manganite perovskites, we mixed the lanthanum strontium manganite perovskites with SDC to develop a semiconductor-ionic nanocomposite functional layer for SOFC. Study results revealed that the composite possessed better conductivity and electrochemical performance than the conventional pure SDC electrolyte.

## 2. Experimental

### 2.1. Materials Synthesis and Physical Characterizations

Both La_0.1_Sr_0.9_MnO_3−δ_ (LSM) with Ce_0.8_Sm_0.2_O_2−δ_ (SDC) materials were synthesized by co-precipitation method. For LSM synthesis, 0.0025 mol La(NO_3_)_3_·6H_2_O (Aladdin Industrial Corporation, Shanghai, China), 0.0225 mol Sr(NO_3_)_2_ (Sinopharm Chemical Reagent Co., Ltd., Shanghai, China), and 0.025 mol Mn(NO_3_)_2_ (Sinopharm Chemical Reagent Co., Ltd., Shanghai, China) were dissolved in 100 mL deionized water. Na_2_CO_3_ (Sinopharm Chemical Reagent Co., Ltd., Shanghai, China) solution (0.5 mol L^−1^, 154 mL) was added to the above solution dropwise. After 2 h stirring, it was left to be aged for 12 h. The precipitation was filtrated by Buchner funnel (Jiangsu Sanaisi Scientific Instrument Corporation, Jiangsu, China), followed by washing with de-ionic water, drying at 120 °C for 2 h, and then calcinated at 1000 °C for 6 h to get the LSM sample. The SDC was synthesized with the similar steps, except for the calcinations condition (800 °C for 4 h).

The LSM-SDC composites were fabricated by grinding the mixture of LSM and SDC powders manually with the agate mortar.

### 2.2. Material Characterizations

X-ray diffraction (XRD) measurement was conducted on Bruker D8 (Cu Kα radiation, λ = 0.154 nm, Bruker Corporation, Karlsruhe, Germany). The sample morphologies and chemical compositions were examined by scanning electron microscope (FE-SEM, JSM7100F, Japan Electron Optics Laboratory Co., Ltd., Tokyo, Japan) equipped with energy-dispersive X-ray (EDX, Japan Electron Optics Laboratory Co., Ltd., Tokyo, Japan) spectrometer.

### 2.3. Electrochemical Measurement

The nickel foam-NCAL (Ni_0.8_Co_0.15_Al_0.05_LiO_2−δ_, Tianjin B&M Science and Technology Joint-Stock Co., Ltd., Tianjin, China) electrodes were prepared by the brush coating-method [32,33]. After being pressed, the thickness of the Ni-NCAL electrode was about 0.4 mm. The Ni-NCAL (anode and cathode) and LSM-SDC (electrolyte layer) were dry-pressed (200–300 MPa pressure) together to obtain the SOFC device. There was no further firing procedures before testing the device to protect the hetero-interfaces between LSM and SDC [19,20]. Fuel cell thickness and effective areas were 0.7 mm and 0.64 cm^2^, respectively.

The electronic load (type IT8512C+, ITECH Electronic Co., Ltd., Nanjing, China) was used to evaluate the fuel cell performance (with air and H_2_ as oxidant and fuel, respectively). Electrochemical impedance spectroscopy (EIS) measurement was conducted by Gamry Ref 3000 (Gamry Instruments, Philadelphia, PA, USA) under OCV (amplitude: 10 mV, frequency range: 1 MHz–0.1 Hz). An equivalent circuit mode LR_Ω_(R_a_Z_a_)((R_b_Z_b_)(R_c_Z_c_) was used to fit the original EIS data by ZView (Copyright 1990–2007 Scribner Associates, Inc., Southern Pines, NC, USA, written by Derek Johson). The electronic conductivities of LSM-SDC composites were measured by Keithley 2400 SourceMeter (Tektronix Corporation, Beaverton, WA, USA).

## 3. Results and Discussion

**Figure 1:**
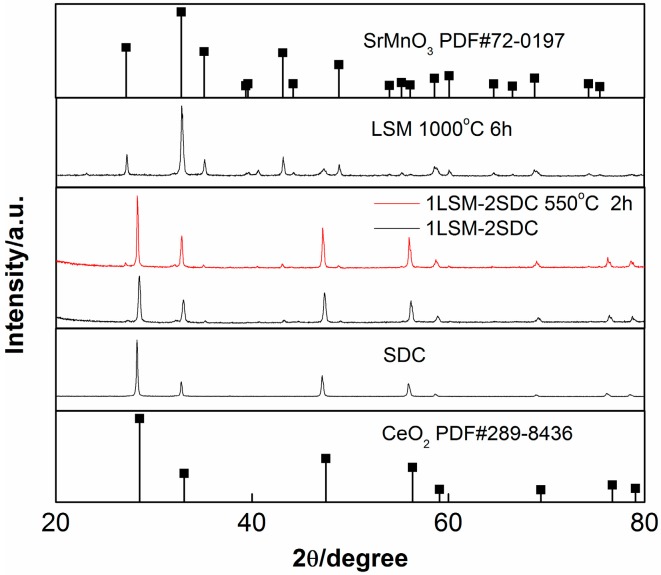
XRD patterns of LSM (La_0.1_Sr_0.9_MnO_3−δ_), SDC (Ce_0.8_Sm_0.2_O_2−δ_) and LSM-SDC composite before and after calciation treatment.

As shown in Figure 1, the characteristic peaks of LSM corresponded to a perovskite SrMnO_3_ structure. The diffraction peaks of prepared SDC material were indexed to standard diffraction pattern for fluorite CeO_2_ structure, indicating that Sm was doped into the crystal lattice of ceria. The major diffraction peaks of the original 1LSM-2SDC composite belonged to SDC. The characteristic peaks of LSM in the pattern of the original 1LSM-2SDC composite were not obvious due to the smaller amount of LSM in the composite. Compared with the 1LSM-2SDC composite material, there was no new phase in the XRD pattern of 1LSM-2SDC, which was heat-treated at 550 °C for 2 h under the air atmosphere, indicating that the composite is thermal stable under 550 °C. The average crystal sizes of SDC and LSM were estimated according to the Debye-Scherrer equation from the peaks at 28.3° and 35.2°, respectively. Both of them were nanoscale, 46 nm for SDC and 37 nm for LSM. The small radius of the materials was beneficial to increase the hetero-interfaces between LSM and SDC. After being heat-treated at 550 °C for 2 h, the average crystal size of SDC increased to about 48 nm, indicating that the SDC is stable under the fuel cell working temperature.

**Figure 2:**
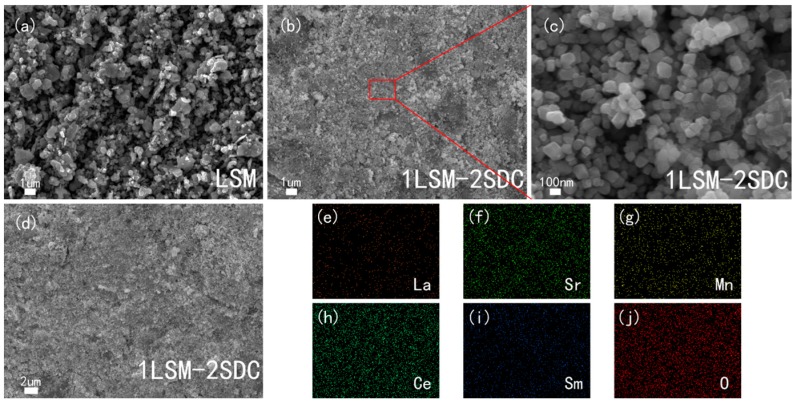
SEM of (**a**) LSM (La_0.1_Sr_0.9_MnO_3−δ_) material, (**b**–**d**) original LSM-SDC (La_0.1_Sr_0.9_MnO_3−δ_-Ce_0.8_Sm_0.2_O_2−δ_) composite (1:2, wt:wt, without co-fire treatment), (**e**–**j**) elemental mappings of the LSM-SDC composite (1:2, wt:wt). The elemental mappings were taken from Figure 2d.

Figure 2a–d showed that the LSM particle is larger than its crystal size obtained from the XRD pattern due to the high calcinating temperature. According to Figure 2b,c the distribution of LSM and SDC in the LSM-SDC composite materials is uniform. It can be further certified by the EDS analysis (Figure 2e–j) of LSM-SDC composite materials (Figure 2d). The uniform distribution of LSM and SDC is beneficial to build continuous networks for ionic conduction.

**Figure 3:**
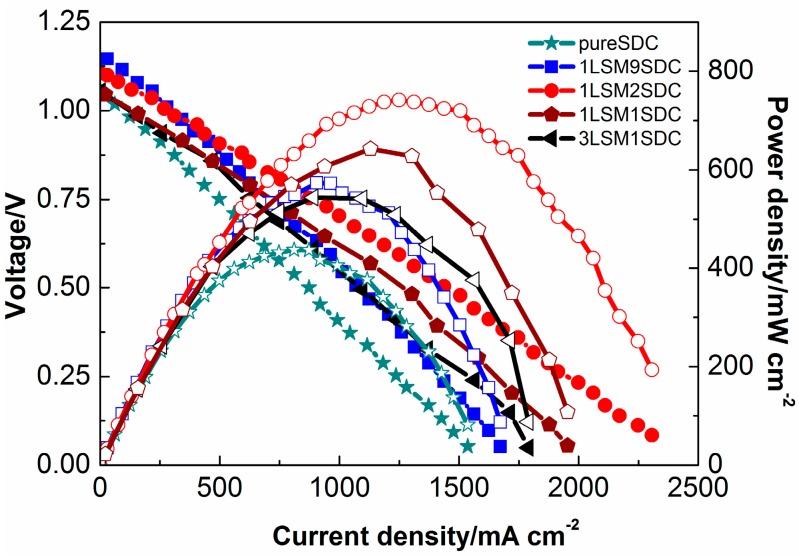
Electrochemical performances (solid marks for I-V curves, empty marks for I-P curves) of SOFCs based on LSM-SDC (La_0.1_Sr_0.9_MnO_3−δ_-Ce_0.8_Sm_0.2_O_2−δ_) layer with various compositions.

Figure 3 revealed that the SOFC performances increased with LSM contents until the weight ratio of LSM to SDC was 1:2. At this weight ratio of 1:2, the P_max_ (maximum power density) 742 mW cm^−2^ has been achieved at 550 °C. Further increasing the content of the LSM caused a decrease in the fuel cell performance. Compared with the pure SDC, the LSM-SDC composite possessed a larger amount of hetero-interfacial region/interface, which could provide fast pathways for ionic conducting [2,14,15,16,17,18]. At the above optimum weight ratio, the ionic grain boundary conductivity was the best due to the highest hetero-interfacial area. The following characterizations will give further confirmation.

LSM is a common SOFC cathode material with excellent electronic conductivity. It is interesting that no short circuit phenomenon occurred for the SOFC using the LSM-SDC composite layer to replace the conventional electrolyte layer with the pure ionic conductor. All of the OCVs were over than 1.0 V. It may be noticed that even in the case with pure doped ceria as the electrolyte for SOFCs, short circuiting problem with low OCVs (below 1.0 V, more often 0.9 V) has been commonly observed [11,34]. Therefore, both electronic and ionic conductivities were investigated for the LSM-SDC composite samples with various weight ratios. Ag paste was brushed onto both sides of the LSM-SDC functional layer in a configuration of Ag/LSM-SDC/Ag for electrical property measurements. The electronic conductivities of LSM-SDC composites were determined by potentiostatic polarization method with the constant voltage of 0.5 V. The test temperature and atmosphere were 550 °C and N_2_, respectively. In the beginning, both electrons and O^2−^ can make contributions to the current. But O^2−^ would be exhausted with time due to no ionic resource from the N_2_ atmosphere, and electrons are then the only charge carriers in LSM-SDC composites when the current reach a steady state (I_s_) and we can get the electronic resistance R_e_ = 0.5/I_s_ [35]. The total resistance of the LSM-SDC composites was measured by EIS under H_2_/air atmosphere. The EIS results were fitted through the equivalent circuit of LR_Ω_(R_a_Z_a_)((R_b_Z_b_)(R_c_Z_c_), where L-inductance, R_Ω_-ohmic resistance, R_a_-ionic grain boundary transportation resistance, R_b_(R_c_)-charge-transfer resistance and Z-constant phase element. The total resistances were given by the following equation.

R_t_ = R_Ω_ + R_a_ (1)

**Figure 4:**
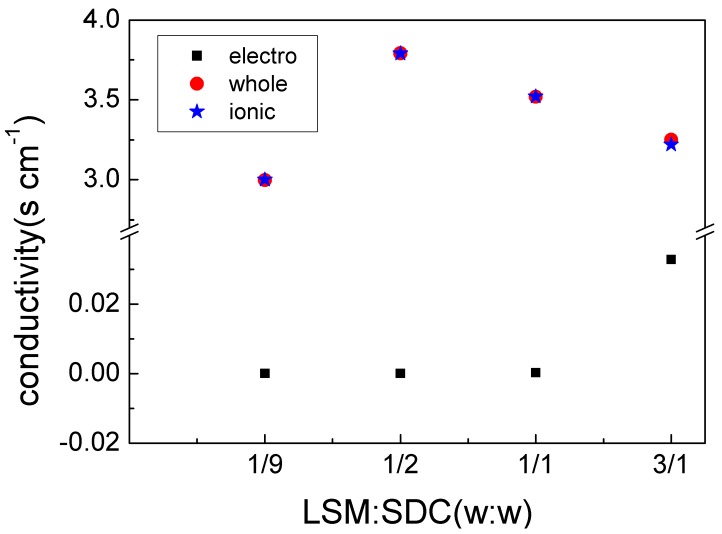
Conductivities for LSM-SDC (La_0.1_Sr_0.9_MnO_3−δ_-Ce_0.8_Sm_0.2_O_2−δ_) composites with various compositions.

The ionic conductivity was calculated by subtracted electronic conductivity from total conductivity. The calculated data are listed in Figure 4, which revealed that the composite electronic conductivity increased with LSM content but still extremely small, indicating a low electronic conductivity owing to the discontinuity of the electronic pathway in the device.

**Figure 5:**
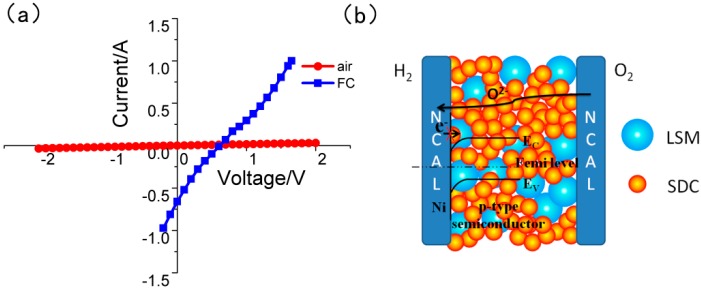
(**a**) I-V curves for the SOFCs with LSM-SDC (La_0.1_Sr_0.9_MnO_3−δ_-Ce_0.8_Sm_0.2_O_2−δ_) with weight ratio of 1:2. Scan rate: 50 mV/s; Test conditions: H_2_/air atmosphere (FC), air/air atmosphere (air), 550 ^o^C; (**b**) Schematic of the Schottky junction in the fuel cell.

Linear scan voltage was supplied on the SOFC device with LSM-SDC (1:2, weight ratio) as the functional layer (Figure 5a). When the anode and cathode of the SOFC were both supplied with air, the I-V curve is linear, indicating that the fuel cell corresponds to a linear resistor under the air atmosphere; when the anode gas was changed into H_2_, the I-V curve becomes non-linear, indicating that there is a diode or rectification effect in the fuel cell device. As a kind of p-type semi-conductor, LSM, close to the anode side, can form a Schottky junction with metal Ni, production of NCAL reduction reaction [36,37]. The built-in electric field, generated by Schottky junction, not only drives the ionic transportation, but also prevents electrons transporting from the anode to cathode (Figure 5b). Therefore, even though there is some electronic conduction in the LSM-SDC composite, it can be prevented by the electronic blocking layer in the device.

**Figure 6:**
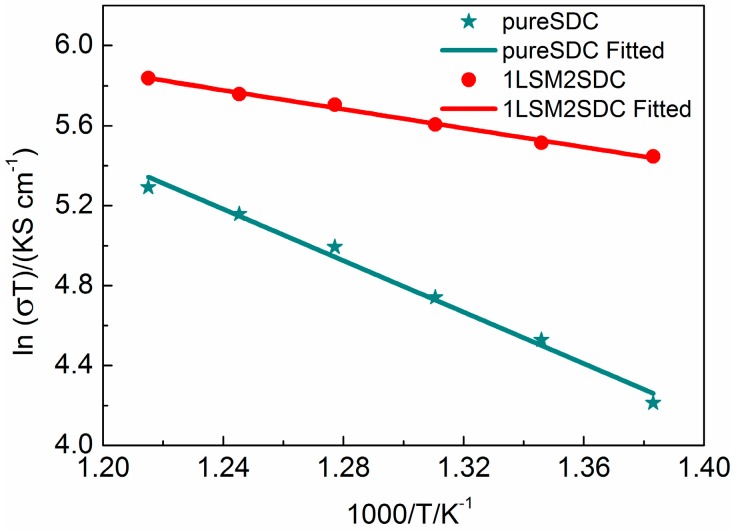
Arrhenius plots of ionic conductivity of LSM-SDC (La_0.1_Sr_0.9_MnO_3−δ_-Ce_0.8_Sm_0.2_O_2−δ_) composite (1:2, weight ratio) and pure SDC material.

Figure 6 shows Arrhenius plots of pure SDC and LSM-SDC (1:2, weight ratio) composite at the temperature from 450 °C to 550 °C. Conductivities were measured by the EIS method in the fuel cell atmosphere. The measured EIS at different temperatures were also fitted with the equivalent circuit of LR_Ω_(R_a_Z_a_)((R_b_Z_b_)(R_c_Z_c_). The conductivity σ can be obtained by

σ = δ/(A(R_Ω_ + R_a_)) (2)

where δ-pallet thickness (0.7 mm), A-pallet surface area (0.64 cm^2^). It is noted that two samples exhibited a linear dependence between 550 and 450 °C. The activation energy can be calculated by the slope of the linear line, which is 0.20 eV for the LSM-SDC composite (1:2, weight ratio) and 0.55 eV for SDC, respectively. This indicates that the LSM-SDC composite possesses possible interfacial conduction with lower activation energy than that of the SDC via bulk conduction of pure SDC [32]. It is consistent with the fuel cell performance in Figure 3.

**Figure 7:**
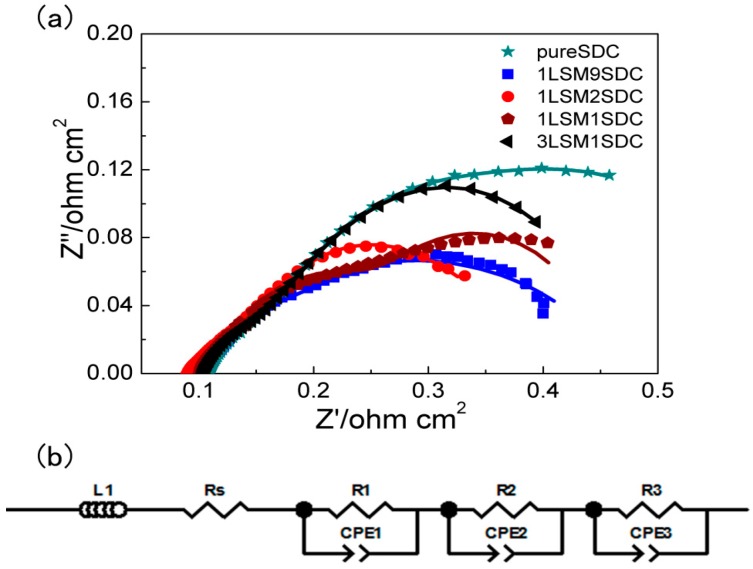
(**a**) EIS of the LSM-SDC (La_0.1_Sr_0.9_MnO_3−δ_-Ce_0.8_Sm_0.2_O_2−δ_) composite series measured at 550 °C, OCV, fuel cell mode; points for experimental data, line for fitting data, (**b**) equivalent circuit.

To further investigate the ionic grain boundary transportation resistance of LSM-SDC composites, the EIS measurement was conducted, and the results were revealed in Figure 7a. The intercept at high frequency reflects ohmic resistance, whereas the diameters of the semicircles at medium and low frequency reflect the ionic grain boundary transportation resistance and charge-transfer resistance, respectively [37,38]. An equivalent circuit mode LR_Ω_(R_a_Z_a_)((R_b_Z_b_)(R_c_Z_c_) (Figure 7b) is still employed to fit the EIS data. The fitting results (Table 1) showed that, compared with SDC (R_a_ = 0.084 Ω·cm^2^), LSM-SDC composites possessed lower ionic grain boundary transportation resistances (R_a_, 0.023–0.060 Ω·cm^2^). At the hetero-interface, such as between perovskite LSM material and fluorite SDC material, atomic reconstruction generates high-mobility plane with a large amount of oxygen vacancies, yielding colossal values of the ionic conductivity [2]. At the weight ratio of 1:2 (LSM:SDC), the R_a_ of LSM-SDC composite was the lowest (0.023 Ω·cm^2^), indicating that this proportion is beneficial to construct more hetero-interfaces. It is consistent with the Arrhenius plots (Figure 6) and the fuel cell performance (Figure 3).

## 4. Conclusions

The semiconductor-ionic LSM-SDC composite materials have been synthesized and assembled the SOFCs successfully. The maximum power density of the assembled SOFC was improved from 437 mW cm^−2^ for SDC electrolyte to 742 mW cm^−2^ for LSM-SDC semiconductor-ionic membrane at 550 °C. The interfaces between the semiconductor LSM and ionic conductor SDC, as the ionic transport highways, significantly improved the ionic conductivity of the SOFCs. The Schottky junction, existing in LSM-SDC membrane device, avoided the electronic short circuiting problem. The influences of the composite weight ratio on the fuel cell performance were investigated to find the optimal proportion of the composite, LSM:SDC = 1:2. The successful application of LSM-SDC composite on the LT-SOFCs provides a new direction for the development of LT-SOFCs.

## Figures and Tables

**Table 1:** Fitting results of EIS data in Figure 7a.

Proportion	R_Ω_ (Ω·cm^2^)	R_a_ (Ω·cm^2^)	R_b_ (Ω·cm^2^)	R_c_ (Ω·cm^2^)	R_b_ + R_c_ (Ω·cm^2^)
Pure SDC	0.11	0.084	0.11	0.31	0.42
1LSM9SDC	0.11	0.060	0.080	0.22	0.30
1LSM2SDC	0.090	0.023	0.096	0.21	0.31
1LSM1SDC	0.10	0.046	0.095	0.22	0.31
3LSM1SDC	0.10	0.052	0.074	0.23	0.31
